# Statement of Retraction

**DOI:** 10.1080/10717544.2022.2110727

**Published:** 2022-08-25

We, the Editors and Publisher of the journal *Drug Delivery* have retracted the following article:

Jing-Bo Hu, Gui-Ling Song, Di Liu, Shu-Juan Li, Jia-Hui Wu, Xu-Qi Kang, Jing Qi, Fei-Yang Jin, Xiao-Juan Wang, Xiao-Ling Xu, Xiao-Ying Ying, Lian Yu, Jian You & Yong-Zhong Du (2017) Sialic acid-modified solid lipid nanoparticles as vascular endothelium-targeting carriers for ischemia-reperfusion-induced acute renal injury, Drug Delivery, 24:1, 1856-1867, DOI: 10.1080/10717544.2017.1410258

Since publication, concerns have been raised about the similarity of Figure 5C to Figure 9A in the following article published in *Carbohydrate Polymers*:

Jing-Bo Hu, Shu-Juan Li, Xu-Qi Kang, Jing Qi, Jia-Hui Wu, Xiao-Juan Wang, Xiao-Ling Xu, Xiao-Ying Ying, Sai-Ping Jiang, Jian You, Yong-Zhong Du (2018) CD44-targeted hyaluronic acid-curcumin prodrug protects renal tubular epithelial cell survival from oxidative stress damage, Carbohydrate Polymers, 193, 268-280, DOI:10.1016/j.carbpol.2018.04.011

and the similarity of Figure 4a to Figure S2 in the following article published in *Theranostics*:

Hu JB, Kang XQ, Liang J, Wang XJ, Xu XL, Yang P, Ying XY, Jiang SP, Du YZ (2017) E-selectin-targeted Sialic Acid-PEG-dexamethasone Micelles for Enhanced Anti-Inflammatory Efficacy for Acute Kidney Injury. Theranostics, 7(8), 2204-2219. doi:10.7150/thno.19571

During the investigation, additional concerns such as lack of ethical approval for animal research and use of incorrect reagents were observed. The authors were unable to provide a satisfactory response to the additional concerns. As verifying the validity of published work is core to the integrity of the scholarly record, we are therefore retracting the article. The corresponding author listed in this publication has been informed.

We have been informed in our decision-making by our policy on publishing ethics and integrity and the COPE guidelines on retractions.

The retraction article will remain online to maintain the scholarly record, but it will be digitally watermarked on each page as ‘Retracted’.

